# Characteristics and response of subretinal hyperreflective material to anti-vascular endothelial growth factor in myopic choroidal neovascularization

**DOI:** 10.1038/s41598-023-32417-7

**Published:** 2023-04-03

**Authors:** Chien-Jung Huang, Yun Hsia, Shih-Wen Wang, I.-Hsin Ma, Mei-Chi Tsui, Kuo-Chi Hung, Tzyy-Chang Ho

**Affiliations:** 1 Department of Ophthalmology, Fu Jen University Hospital, New Taipei City, Taiwan; 2 Department of Ophthalmology, College of Medicine, National Taiwan University Hospital, National Taiwan University, No. 7, Chung-Shan S. Rd., Taipei City, 10002 Taiwan; 3 Department of Ophthalmology, Shuang Ho Hospital, Taipei Medical University, New Taipei City, Taiwan; 4 Universal Eye Clinic, Taipei, Taiwan; 5 Department of Ophthalmology, National Taiwan University Biomedical Park Hospital, National Taiwan University Hospital Hsinchu Branch, HsinChu County, Zhubei City, Taiwan

**Keywords:** Biomarkers, Risk factors

## Abstract

This retrospective study evaluated the characteristics and response of subretinal hyperreflective material (SHRM) to anti-vascular endothelial growth factor (VEGF) treatment in eyes with myopic choroidal neovascularization (CNV). The visual acuity (VA) was assessed at 3, 6, and 12 months after initiating anti-VEGF treatment in 116 patients (119 eyes) with SHRM and myopic CNV. Multimodal imaging, including color fundus photography, fluorescein angiography (FA), and optical coherence tomography angiography (OCT-A), were performed. We compared type 2 neovascularization (NV) (n = 64), subretinal hyperreflective exudation (SHE) (n = 37), NV with hemorrhage (n = 15), and fibrosis (n = 3). The type 2 NV group, and NV with hemorrhage groups showed significant VA improvement after 12 months of treatment (p < 0.05 in both groups); the SHE group failed to show improvement (p = 0.366). All groups showed a significant reduction in central foveal thickness after 12 months of treatment (all p < 0.05). The SHE group had a significantly higher incidence of interrupted ellipsoid zone than the other groups (p < 0.05). Myopic CNV can present as SHRM on OCT-A. Visual prognoses vary in different SHRM types. OCT-A and FA may help predict the outcomes of different subtypes of myopic CNV. SHE is predictive of outer retinal layer atrophy in patients with various SHRM types.

## Introduction

Myopia, defined as a spherical equivalent refractive error of < − 0.5 D in the least myopic eye, affects 28% of the population worldwide^1^. In the east and southeast Asian regions, such as Taiwan and Japan, myopia occurs in 90% of elementary school children^2–4^. In Asian countries, the prevalence of high myopia (SE < − 6D) is approximately 7.9–16.6%, and that of very high myopia (SE < − 10D) is 0.08–0.92%^5,6^.

Macular choroidal neovascularization (CNV) is the main cause of visual impairment in patients with high myopia^7^. On examination, myopic CNV appears as a yellow-greyish patch, which is occasionally accompanied by active hemorrhage. On fluorescein angiography (FA), CNV appears as early hyperfluorescence with late leakage and staining in the late phase of fluorescein angiography, whereas on sturctual optical coherence tomography it can appear as a hyperreflective material located external to the retina and internal to the retinal pigmented epithelium^8,9^. This hyperreflective material has been termed “subretinal hyperreflective material (SHRM)” in previous neovascular age-related macular degeneration (AMD) studies^10^. SHRM can be further classified into neovascular tissue, subretinal hyperreflective exudation (SHE), hemorrhage, and fibrosis according to the fundus photography, FA, and OCT-A findings^11,12^.

Anti-vascular endothelial growth factor (VEGF) therapy is the first-line treatment for myopic CNV^13^. The long-term visual prognosis of myopic CNV without treatment is poor^14,15^. The RADIANCE and MYRROR studies have demonstrated significant visual improvement in myopic CNV after treatment with ranibizumab and aflibercept^16–21^.

Previous studies on AMD have attempted to clarify the correlation between the visual outcome and different types of SHRM^11,12^. Studies have revealed that, unlike in neovascular AMD or diabetic macular edema, a small amount of intravitreal injection can show sustained and adequate efficacy in the treatment of myopic CNV^22,23^. A minimum number of intravitreal ranibizumab injections in eyes with myopic CNV are associated with good visual outcomes and a 5-year visual benefit^24^. Nevertheless, some patients with myopic CNV who received anti-VEGF treatment experienced poor improvement in visual acuity (VA) and macular atrophy.

The role of SHRM after anti-VEGF therapy, especially in terms of interrupted ellipsoid zone and macular atrophy formation, in patients with myopic CNV has not been studied. Therefore, to clarify the different treatment outcomes of anti-VEGF according to different SHRM patterns in myopic CNV, we conducted a multimodal imaging study to investigate the characteristics and fate of SHRM after anti-VEGF treatment in patients with myopic CNV.

## Results

We enrolled 119 eyes of 116 patients with SHRM on OCT. These patients were followed up for at least 12 months. The baseline characteristics are shown in Table 1.

**Table 1:** Baseline characteristics of all study eyes.

Total eyes	119
Right (%)	55 (46.2)
Left (%)	64 (53.8)
Number of patients	114
Men (%)	31 (27.2)
Women (%)	83 (72.8)
Age, mean ± SD, years (range)	60.4 ± 12.1 (33–94)
Component of SHRM	
Type 2 Neovascularization (%)	64 (53.8%)
Fibrosis (%)	3 (2.5%)
Subretinal hyperreflective exudation (%)	37 (31.1%)
Haemorrhage (%)	15 (12.6%)
Axial length, mean ± SD, mm (range)	29.57 ± 1.63 (33.72–26.69)
Anti-VEGF used (eyela/lucentis)	12/107

*SD* standard deviation, *SHRM* subretinal hyperreflective material, *VEGF* vascular endothelial growth factor.

**Figure 1:**
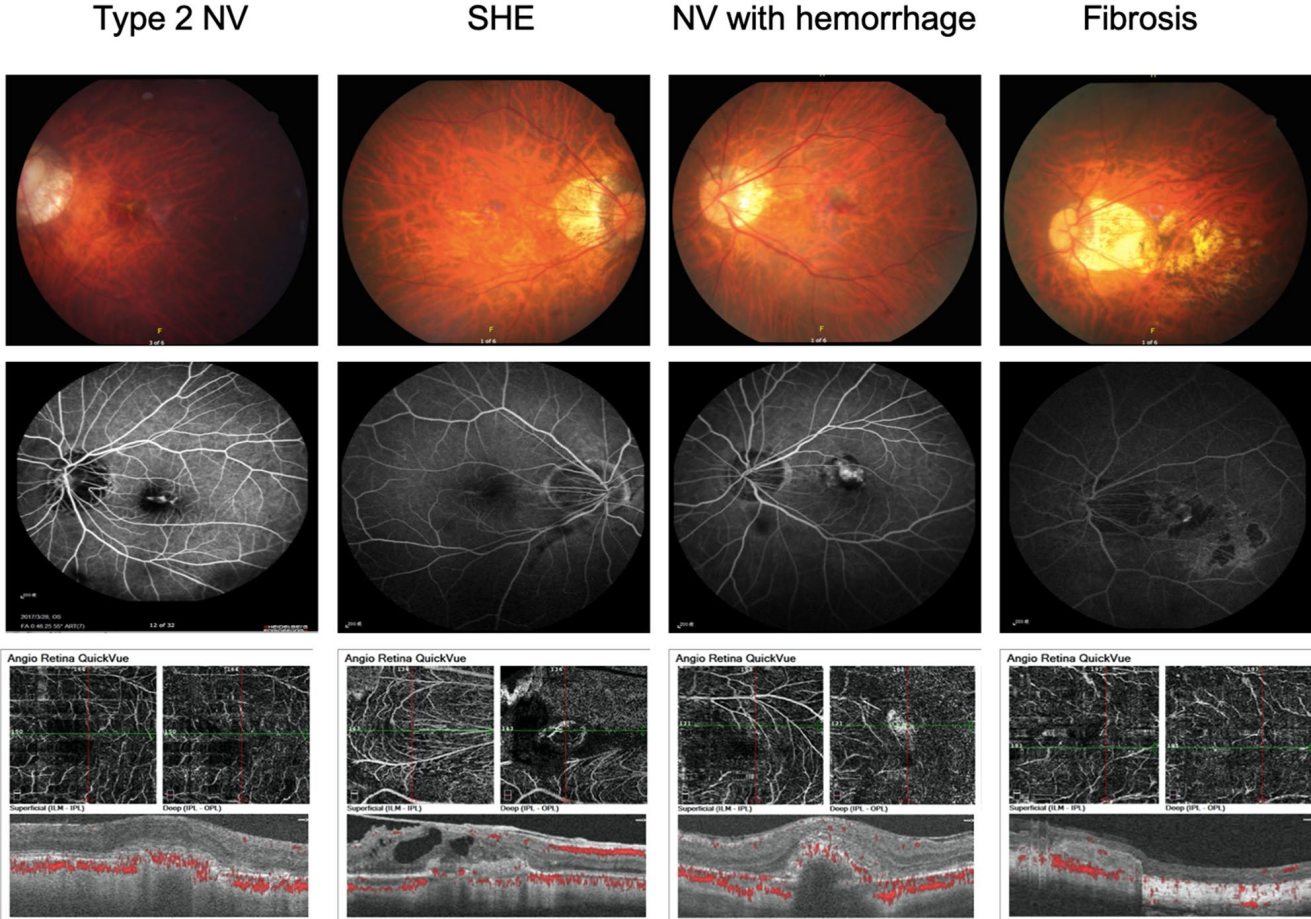
Color fundus photographs and fluorescein angiography and OCT angiography images of different SHRM. *SHRM* subretinal hyperreflective material, *SHE* subretinal hyperreflective exudation, *NV* neovascularization, *OCT* optical coherence tomography.

Among the 119 included eyes, 55 were right eyes, and 64 were left eyes. Among the 116 included patients, 31 were men, and 83 were women. The age of these patients ranged from 33 to 94 years (mean age 60.4 ± 12.1 years). In terms of SHRM type, there were 64 cases of type 2 NV, 37 cases of SHE, 15 cases of hemorrhage, and three cases of fibrosis. The morphological pictures were demonstrated in Fig. 1.

The criteria used to classify SHRM were based on those used in previous studies^25^. The characteristics of the different SHRM groups are summarized in Table 2.

**Table 2:** Differences in the characteristics of the eyes according to the SHRM components.

SHRM component	Type 2 NV	Fibrosis	SHE	Haemorrhage
Number of eyes	64	3	37	15
Sex, male (%)	20 (31.3)	2 (66.7)	6 (16.2)	6 (40.0)
Age, years (mean ± SD)	60.4 ± 11.5	57.3 ± 14.5	61.6 ± 10.6	57.6 ± 14.2
logMAR visual acuity at baseline (mean ± SD)	0.74 ± 0.45	1.23 ± 0.21	0.80 ± 0.34	0.80 ± 0.32
Mean central foveal thickness at baseline (µm, mean ± SD)	295.30 ± 79.91	237.33 ± 27.79	291.32 ± 67.32	350.73 ± 114.66
Axial length (mm, mean ± SD)	29.7 ± 1.7	28.47 ± 2.4	29.7 ± 1.8	29.0 ± 1.0
Presence/absence of SHRM at 12 months	54/10 (84.4%)	3/0 (100%)	30/7 (81.1%)	12/3 (80.0%)
Disrupted/intact ellipsoid zone at 12 months	27/37 (42.2%)	3/0 (100%)	25/12 (67.6%)	7/8 (46.7%)
Presence/absence of IRF or SRF at 12 months	6/58 (9.4%)	0/3 (0%)	4/33 (10.8%)	4/11 (26.7%)
Presence/absence of atrophy	8/56 (12.5%)	3/0 (100%)	8/29 (21.6%)	1/14 (6.7%)
Number of injections (mean ± SD)	2.54 ± 1.15	2	2.60 ± 1.06	2.60 ± 0.91

*SHRM* subretinal hyperreflective material, *SD* standard deviation, *IRF* intra-retinal fluid, *SRF* subretinal fluid, *NV* neovascularization, *SHE* subretinal hyperreflective exudation.

The baseline characteristics, including age, sex, axial length, and the number of injections in 1 year, were comparable among the four groups. However, the fibrosis group had a significantly poorer baseline VA, and the hemorrhage group had a higher CFT than the other groups.

The mean logMAR VA at baseline was 0.74 ± 0.45, 1.23 ± 0.21, 0.80 ± 0.34, and 0.80 ± 0.32 in the type 2 NV, fibrosis, SHE, and hemorrhage groups, respectively. All patients received anti-VEGF treatment, and further treatment was administered if SRF or IRF persisted. The mean logMAR VA at 3, 6, and 12 months were 0.63 ± 0.57, 0.62 ± 0.55, and 0.60 ± 0.57, respectively, in the type 2 NV group; 0.78 ± 0.44, 0.83 ± 0.55, and 0.82 ± 0.55, respectively, in the SHE group; and 0.52 ± 0.51, 0.53 ± 0.51, and 0.46 ± 0.46, respectively, in the hemorrhage group. At 3, 6, and 12 months, VA in patients with type 2 NV and hemorrhage improved significantly from the baseline VA (p < 0.05) (Table 3).

**Table 3:** Visual acuity and central foveal thickness according to the SHRM components.

	Type 2 NV	SHE	Haemorrhage
LogMAR visual acuity at baseline (mean ± SD)	0.74 ± 0.45	0.80 ± 0.34	0.80 ± 0.32
LogMAR visual acuity at 3 months (mean ± SD)	0.63 ± 0.57*	0.78 ± 0.44	0.52 ± 0.51*
LogMAR visual acuity at 6 months (mean ± SD)	0.62 ± 0.55*	0.83 ± 0.55	0.53 ± 0.51*
LogMAR visual acuity at 12 months (mean ± SD)	0.60 ± 0.57*	0.82 ± 0.55	0.46 ± 0.46*
Mean central foveal thickness at baseline (µm, mean ± SD)	295.29 ± 79.91	291.3 ± 67.32	350.73 ± 114.67
Mean central foveal thickness at 3 months (µm, mean ± SD)	260.54 ± 59.20*	239.85 ± 61.35*	266.93 ± 44.14*
Mean central foveal thickness at 6 months (µm, mean ± SD)	254.97 ± 43.91*	233.51 ± 38.43*	259.13 ± 44.46*
Mean central foveal thickness at 12 months (µm, mean ± SD)	252.52 ± 50.87*	227.11 ± 32.76*	268.47 ± 50.08*

*Compared with baseline: p < 0.05.

*SHRM* subretinal hyperreflective material, *SD* standard deviation, *NV* neovascularization, *SHE* subretinal hyperreflective exudation.

**Figure 2:**
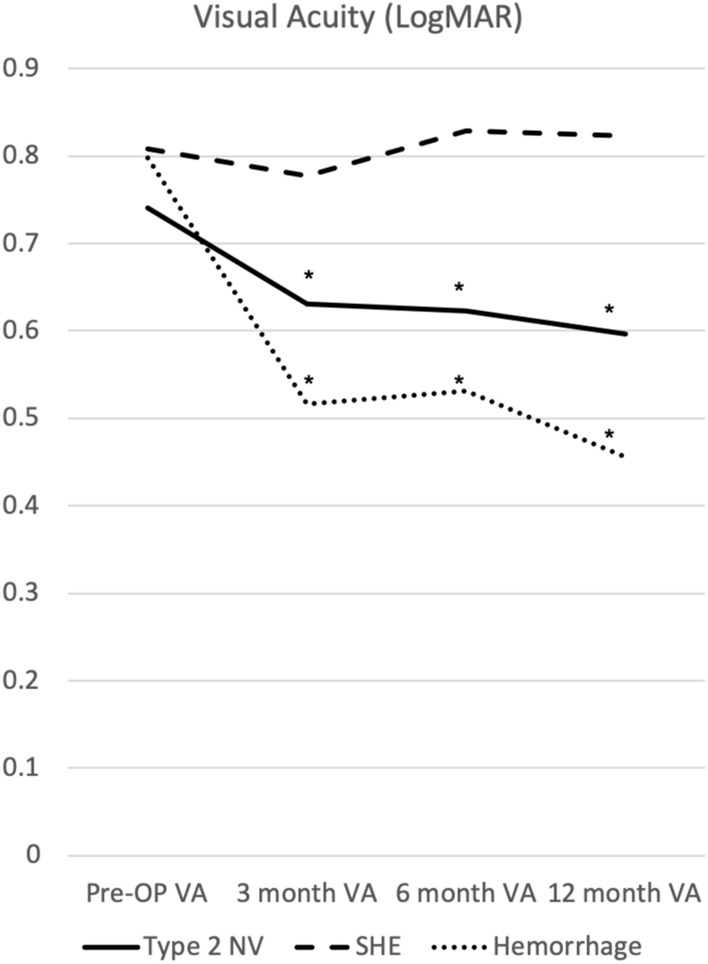
Visual acuity according to different SHRM types after 1 year of treatment. *p < 0.05 compared with baseline visual acuity. *SHRM* subretinal hyperreflective material, *OP* operation, *VA* visual acuity, *NV* neovascularization, *SHE* subretinal hyperreflective exudation.

**Figure 3:**
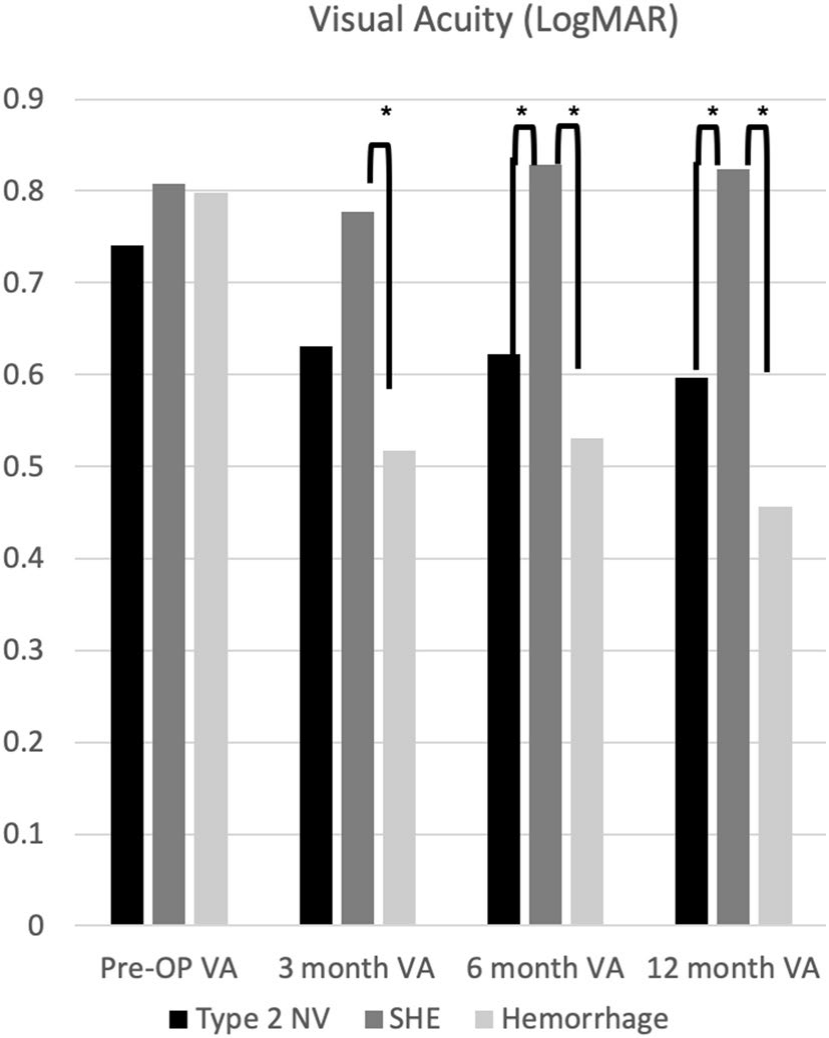
Visual acuity according to different SHRM types after 1 year of treatment. *p < 0.05 compared with baseline visual acuity. *SHRM* subretinal hyperreflective material, *OP* operation, *VA* visual acuity, *NV* neovascularization, *SHE* subretinal hyperreflective exudation.

However, VA failed to improve significantly at 3, 6, and 12 months in patients with SHE (Fig. 2). The VA at baseline was not significantly different among type 2 NV, SHE, and hemorrhage groups. However, after treatment for 3 months, the hemorrhage group showed a significantly better logMAR VA than the SHE group (p < 0.05). Moreover, after treatment for 6 and 12 months, the type 2 NV and hemorrhage groups showed significantly better logMAR VA than the SHE group (p < 0.05) (Fig. 3).

**Figure 4:**
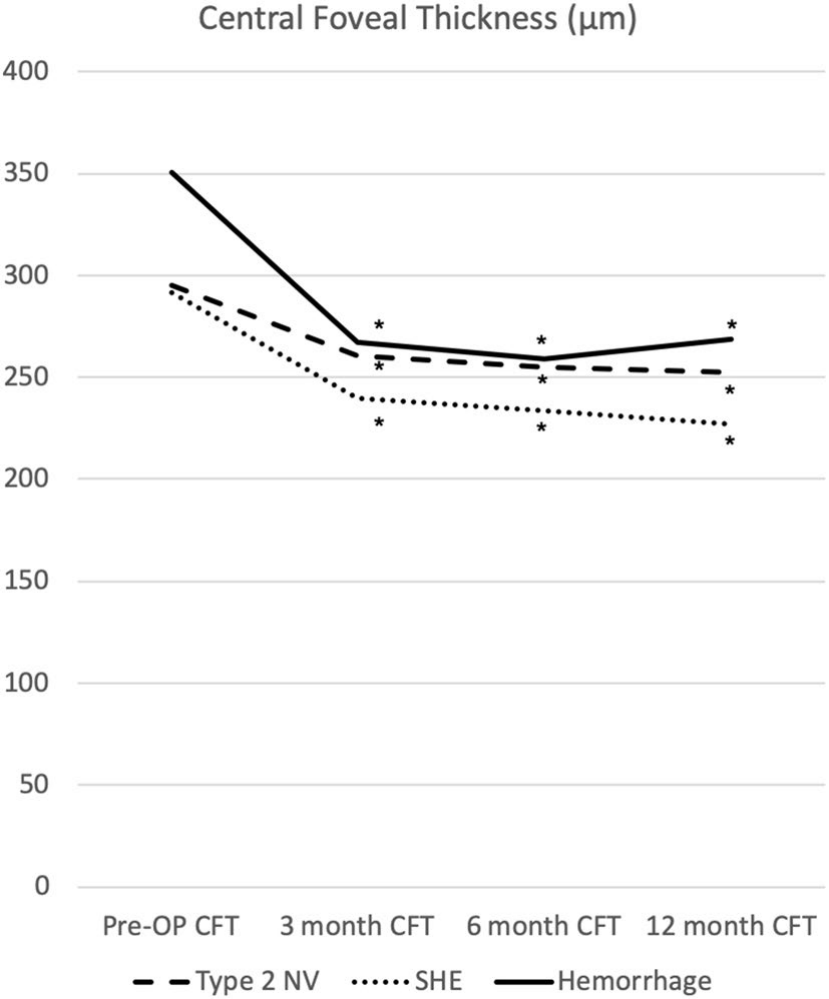
Central foveal thickness according to different SHRM types after 1 year of treatment. *p < 0.05 compared with baseline visual acuity. *SHRM* subretinal hyperreflective material, *OP* operation, *CFT* central foveal thickness, *NV* neovascularization, *SHE* subretinal hyperreflective exudation.

The mean CFT at baseline was 295.29 ± 79.91 µm, 291.3 ± 67.32 µm, and 350.73 ± 114.67 µm in the type 2 NV, SHE, and hemorrhage groups, respectively. During the follow-up at 3, 6, and 12 months, the mean CFT was 260.54 ± 59.20 µm, 254.97 ± 43.91 µm, and 252.52 ± 50.87 µm, respectively, in the type 2 NV group; 239.85 ± 61.35 µm, 233.51 ± 38.43 µm, and 227.11 ± 32.76 µm, respectively, in the SHE group; and 266.93 ± 44.14 µm, 259.13 ± 44.46 µm, and 268.47 ± 50.08 µm, respectively, in the hemorrhage group. During follow-up at 3, 6, and 12 months, CFT in the type 2 NV, SHE, and hemorrhage groups all showed a significant reduction compared with the baseline values (p < 0.05) (Fig. 4).

**Figure 5:**
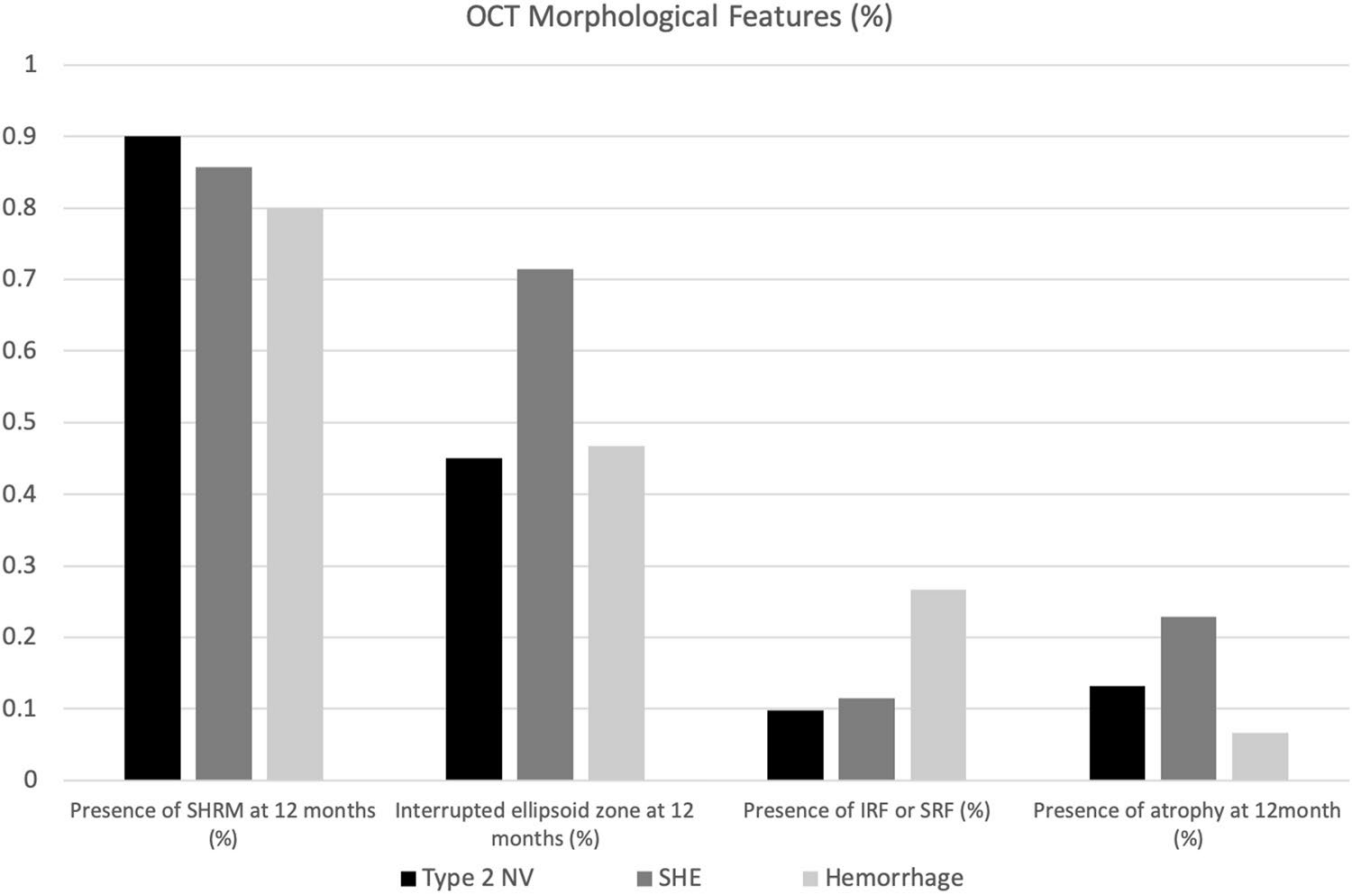
Morphological features on OCT according to different SHRM types after 1 year of treatment. *OCT* optical coherence tomography, *SHRM* subretinal hyperreflective material, *IRF* intra-retinal fluid, *SRF* subretinal fluid, *NV* neovascularization, *SHE* subretinal hyperreflective exudation, *OCT* optical coherence tomography.

In terms of the OCT morphological features in the different SHRM groups (Table 4), there were no differences in the resolution of SHRM, presence or absence of IRF or SRF, and presence of atrophy at 12 months. The SHE group had a higher proportion of patients with interrupted ellipsoid zone at 12 months than the type 2 NV group (p < 0.05) (Fig. 5).

**Table 4:** OCT morphological features in different SHRM groups.

p-value	Presence of SHRM at 12 M	Interrupted ellipsoid zone at 12 M	Presence of IRF or SRF at 12 M	Presence of atrophy at 12 M
Type 2 NV, SHE, haemorrhage	0.873	0.045*	0.173	0.295
Type 2 NV vs. SHE	0.670	0.013*	0.816	0.226
Type 2 NV vs. haemorrhage	0.680	0.752	0.070	0.522
SHE vs. haemorrhage	0.929	0.160	0.151	0.196

*Compared with baseline: p < 0.05.

*OCT* optical coherence tomography, *NV* neovascularization, *SHE* subretinal hyperreflective exudation, *SHRM* subretinal hyperreflective material, *M* month, *IRF* intra-retinal fluid, *SRF* subretinal fluid.

Further analysis of the atrophy and non-atrophy subgroups at 12 months after treatment revealed that older age and poor VA at baseline were associated with a significantly higher risk of atrophy after 12 months of treatment. Other factors, including sex, axial length, mean CFT at baseline, anti-VEGF use, or the number of injections, failed to show significant differences between the atrophy and non-atrophy groups (Table 5).

**Table 5:** Characteristics of the atrophy and non-atrophy groups.

	Atrophy	Non-atrophy	p-value
Age (years, mean ± SD)	65.52 ± 12.06	59.12 ± 11.43	0.014*
Sex (%, male/female)	0.421	0.253	0.069
Axial length (mm, mean ± SD)	29.74 ± 1.90	29.54 ± 1.55	0.348
LogMAR visual acuity at baseline (mean ± SD)	1.023 ± 0.329	0.723 ± 0.384	0.001*
Mean central foveal thickness at baseline (µm, mean ± SD)	315.42 ± 120.36	299.57 ± 73.43	0.224
Anti-VEGF used (%, Eyela/Lucentis)	0.105	0.105	0.500
Number of injections (mean ± SD)	2.58 ± 0.84	2.55 ± 1.12	0.453

*Compared with baseline: p < 0.05.

*SD* standard deviation, *VEGF* vascular endothelial growth factor.

## Discussion

High myopia, which is defined as a spherical equivalent < − 6.0 D or axial length > 26.0 mm, is often accompanied by pathologic changes in the eye, and 10% of patients with pathologic myopia develop CNV over 10–11 years. Pathologic myopia is the second most common cause of CNV following AMD^26^.

Anti-VEGF is now considered the first-line treatment for myopic CNV. The efficacy and safety of ranibizumab were demonstrated in the Phase III RADIANCE and Phase II REPAIR clinical trials, which showed substantial vision gains after treatment with ranibizumab. Over the post-RADIANCE observation period, 83% of patients required no further treatment for myopic CNV, whereas 10% experienced myopic CNV recurrences^19^. A study on intravitreal injection of bevacizumab with a 6-year follow-up demonstrated that bevacizumab could prevent the development of macular atrophy, which is important for long-term visual outcomes in eyes with active CNV, and the final BCVA at 6 years was significantly correlated with the size of the CNV-related macular atrophy, baseline BCVA, and CNV size^21^.

A previous study revealed that SHRM is related to VA in patients with AMD^10,12^. In AMD, the presence of central SHRM, highly reflective SHRM, well-defined SHRM borders, IRF, and thick SHRM predicted poor vision 1 year after IVB treatment^27^. In another study on the association between the morphological features of SHRM and VA in eyes with AMD, a layered appearance, increased reflectivity, large size, and hyperreflective SHRM spots correlated with poor VA at the 12- and 24-week follow-ups. In patients with AMD, the baseline SHRM characteristics can help practitioners predict the visual and morphological prognosis and guide therapy^11^.

This is the first study to describe the types of SHRM in patients with myopic CNV. Furthermore, SHRM can be classified into type 2 NV, SHE, fibrosis, and hemorrhage. Different types of SHRM may have different characteristics and respond differently to anti-VEGF treatment in patients with neovascular AMD^12^. Therefore, this study also aimed to evaluate the response to anti-VEGF treatment and VA recovery in patients with myopic CNV who have different types of SHRM.

The different types of SHRM can be distinguished from each other using different imaging techniques. Type 2 NV appears as a more prominent leakage and staining of the CNV than the SHE on FA. Fibrosis appears as a yellow-white patch on fundus microscopy and late staining on FA. Hemorrhage is easily distinguished from other SHRM types using fundus microscopy. However, FA has the disadvantage of contrast allergy and may pose a threat to patients with renal impairment. With the development of OCT-A, we can easily classify SHRM, even without FA^25^. A primary flow and neovascular membrane are observed in type 2 NV, whereas blood flow is not observed in SHE. Hemorrhage has a prominent blocking effect on the choriocapillaris. As for SHRM in neovascular AMD, OCT-A proved to be a powerful tool for classifying SHRM in patients with myopic CNV.

After initial treatment with anti-VEGF for 12 months, eyes with type 2 NV and hemorrhage showed significant improvement in VA at 3, 6, and 12 months compared with the baseline VA. However, no significant improvement was noted at 3, 6, and 12 months in the SHE group. The SHE group also showed a significantly poorer VA at 6 and 12 months than the type 2 NV and hemorrhage groups. This result is compatible with our finding on the OCT morphological features. The SHE group had a significantly higher incidence of interrupted ellipsoid zone after 1 year of treatment than the other groups. The failure of anatomical recovery of the ellipsoid zone leads to poor VA recovery in patients with SHE. Interestingly, this result was different from that of a study on SHRM in patients with neovascular AMD, which showed that SHE in patients with neovascular AMD is related to good visual recovery and a high incidence of restoration of the ellipsoid zone^12^. The reason for this difference may be the different etiology and chronicity of myopic CNV and neovascular AMD. In patients with AMD, vision loss is often gradual, and SHE is followed by resolution of NV. However, in patients with myopic CNV, hemorrhage and type 2 NV acutely affect VA. These patients would receive anti-VEGF sooner than patients with SHE and the other groups of SHRM. This may be the reason why patients with myopic CNV in the SHE group had poor VA.

In the different groups of SHRM, the intravitreal injection was administered if there were OCT findings of persistent or recurrent IRF or SRF. However, the number of injections administered among the different groups of SHRM was not significantly different.

Different OCT morphological feature improvements were observed after treatment for 12 months for different SHRM types (Table 4). There were no differences in the resolution of SHRM, IRF or SRF, and the risk of macular atrophy in the type 2 NV, SHE, and hemorrhage groups. The major difference in the OCT morphology in different SHRM types was the restoration of the interrupted ellipsoid zone at 12 months. The rate of interrupted ellipsoid zone after treatment with anti-VEGF for 12 months in the SHE group (67.6%) was significantly higher than that in the type 2 NV (42.2%) and hemorrhage (46.7%) groups. CFT reduced after 12 months of treatment in the type 2 NV, SHE, and hemorrhage groups, and slow restoration of the ellipsoid zone in the SHE group led to the failure in improving the VA.

We also analyzed the different characteristics of the patients in the atrophy and non-atrophy groups. We found that older age, poor VA at baseline, and SHE were the factors that increased the risk of atrophy. This may be due to these factors being related to a low reserve of RPE function and a disposition toward the loss of RPE after treatment with anti-VEGF. The axial length, CFT, and the number of injection failures may also predict the risk of atrophy.

The limitations of this study were its retrospective design, short follow-up time, and the use of different anti-VEGF antibodies. Further prospective studies are needed to confirm these results.

In conclusion, color fundus photography, FA, and OCT-A helped us identify different types of SHRM in patients with myopic CNV. These findings may enable a better prediction of the visual outcome after the treatment of myopic CNV with anti-VEGF.

## Materials and methods

We conducted a retrospective analysis of 119 eyes of 116 patients with myopic CNV who received anti-VEGF treatment. The patients were followed up for at least 12 months after the first anti-VEGF treatment. All patients were followed up at the Department of Ophthalmology, National Taiwan University Hospital (NTUH), between October 2007 and February 2014. The retrospecitve study was approved by the Institutional Review Board of National Taiwan University Hospital(202007059RIND) and the study was conducted in accordance with the tenets of the Declaration of Helsinki. Informed consent was waived by the Institutional Review Board of National Taiwan University Hospital due to retrospective nature of study.

All patients underwent slit lamp examination, Snellen best-corrected VA (BCVA) testing, color fundus photography, FA, spectral-domain OCT, and OCT-A. Since the RTVue XR Avanti SD-OCT platform is launched in 2003. Before RTVue XR Avanti was laucnhed, the patient received spectral domain struture OCT and fluorescein angiography. The VA results were converted to logMAR values for further statistical analysis.

The inclusion criteria were eyes with myopic CNV, observation of foveal SHRM on FA and OCT-A, initial (pre-treatment) VA of 20/400 or better, and a follow-up period of at least 1 year. Patients with eye diseases that affect VA, including polypoidal choroidal vasculopathy, diabetic retinopathy, retinal detachment, and glaucoma, and those who previously received treatment for myopic CNV were excluded.

Myopic CNV was diagnosed using color fundus photography, FA, and OCT. These patients received intravitreal injections of ranibizumab (0.5 mg/0.05 ml) or aflibercept (2.0 mg/0.05 ml) via a 30-gauge needle through the pars plana. All patients underwent monthly follow-up evaluations and received additional injections as needed. Repeat injections were administered to patients with persistent or recurrent intra-retinal fluid (IRF) or subretinal fluid (SRF).

BCVA was measured before treatment and at 3, 6, and 12 months after the first injection. The OCT morphological features, including central foveal thickness (CFT), the morphology of the ellipsoid zone, the presence or absence of IRF or SRF, and the presence or absence of subfoveal atrophy at 12 months, were collected from the patients’ medical records. Atrophic changes included the loss of retinal pigment epithelium (RPE) and the outer retinal segment on OCT.

The OCT-A system used in our study (RTVue XR Avanti, Optovue Inc., Fremont, CA, USA) was a spectral domain-OCT device with an A-scan rate of 70,000 scans per second, a light source centered at 840 nm, and a bandwidth of 45 nm to obtain split-spectrum amplitude-decorrelation angiography images. The component and classification were judged according to the criteria described previously^25^. If multiple components of SHRM were noted on OCT, we classified them according to the SHRM in the foveal center. Myopic SHRM was classified into four groups: type 2 neovascularization (NV), SHE, fibrosis, and hemorrhage. All patients received treatment with anti-VEGF agents.

The primary outcome measure was the mean change in BCVA after treatment for 12 months in the different groups of SHRM. The OCT morphological features, including CFT, the integrity of the ellipsoid zone, the presence or absence of IRF or SRF, and the presence or absence of atrophy, were analysed in the four groups. The interrupted ellipsodie zone is the continuous signal noted on structured OCT showed discontinuity.

After testing the normality of the data, we used the paired *t*-test to analyze the changes in VA and CFT after treatment in the four groups. The OCT morphological features were compared among the different groups using the chi-square test. P < 0.05 was considered statistically significant.

## Data Availability

The datasets generated during and/or analyzed during the current study are available from the first author (Tzyy-Chang Ho) on reasonable request.
